# Maternal psychopathic traits prospectively predict youth callous-unemotional traits

**DOI:** 10.3389/frcha.2026.1894994

**Published:** 2026-07-22

**Authors:** Nicholas D. Thomson, Jessica J. James

**Affiliations:** 1 Department of Surgery, Virginia Commonwealth University, Richmond, VA, United States; 2 Departments of Psychology and Psychiatry, Virginia Commonwealth University, Richmond, VA, United States

**Keywords:** callous-unemotional traits, conduct disorder, limited prosocial emotions, psychopathy, psychopathy facets

## Abstract

**Background:**

Callous-unemotional (CU) traits identify a subgroup of youth with conduct disorder (CD) at heightened risk for persistent antisocial behavior and poor long-term outcomes. Although parental psychopathic traits have been associated with offspring CU traits, less is known about whether specific psychopathic facets uniquely predict adolescent CU trajectories beyond baseline CU traits, particularly among youth diagnosed with CD.

**Methods:**

The present study examined whether maternal psychopathic traits were prospectively associated with higher youth CU traits over a 12-month period in a sample of adolescents diagnosed with CD. The sample included 99 youth-caregiver dyads (*N* = 198 individuals). Youth were between 13 and 17 years of age (*M* = 15.52, *SD* = 1.31). Maternal psychopathic traits were assessed using the Self-Report Psychopathy Scale-Short Form (SRP-SF), including total psychopathy and interpersonal, affective, lifestyle, and antisocial facets. Youth CU traits were assessed using the Inventory of Callous-Unemotional Traits (ICU) at baseline and at 12-month follow-up. Analyses controlled for baseline CU traits, youth age, sex, race, and maternal age.

**Results:**

Baseline CU traits were associated with higher CU traits at 12-month follow-up (*r* = .39). Maternal affective psychopathic traits were prospectively associated with higher adolescent CU traits, whereas interpersonal, lifestyle, and antisocial psychopathic traits were not significant predictors. Associations between maternal affective psychopathic traits and adolescent CU traits were stronger among girls than boys.

**Conclusions:**

Maternal affective psychopathic traits were prospectively associated with adolescent CU traits beyond baseline CU traits. These findings suggest that caregiver personality characteristics, particularly affective psychopathic traits, represent important familial risk markers associated with adolescent CU trajectories and highlight the value of examining caregiver personality characteristics in future developmental research.

## Introduction

Callous-unemotional (CU) traits, which are characterized by reduced empathy, shallow emotional expression, and diminished guilt (1), identify a clinically meaningful subgroup of youth with conduct disorder (CD) who exhibit severe and persistent antisocial behavior (2, 3). The importance of this high-risk subgroup has been internationally recognized through the inclusion of CU traits as the Limited Prosocial Emotions (LPE) specifier for CD in major diagnostic classification systems (1, 4). Recent research found that approximately 19% of youth diagnosed with CD met criteria for the LPE specifier (5). Youth with elevated CU traits are more likely to engage in proactive aggression, show reduced responsiveness to punishment, and experience poorer long-term outcomes, including increased risk for adult psychopathy (2, 6). Although early family influences on CU traits are well documented, less research has examined whether caregiver characteristics continue to shape CU trajectories during adolescence in diagnosed samples.

Caregiver characteristics have emerged as an important area of investigation in developmental models of CU traits. Research increasingly suggests that stable caregiver personality characteristics contribute to the broader familial context in which children develop and may therefore be associated with children's socioemotional functioning across childhood and adolescence (7, 8). At the same time, substantial evidence indicates that CU traits are moderately to highly heritable, with genetic influences accounting for a significant portion of individual differences in CU traits across development (9, 10). Importantly, however, contemporary developmental models do not view genetic and environmental influences as competing explanations. Rather, environmental experiences may modify the expression of genetic liability. For example, genetically informed studies suggest that characteristics of the caregiving environment can moderate genetic risk for CU traits (9, 11, 12). Thus, associations between caregiver characteristics and youth CU traits may reflect shared genetic liability, environmental influences, passive gene-environment correlation, or interactions between genetic vulnerability and caregiving contexts (13). Identifying caregiver characteristics associated with CU traits therefore remains important because such characteristics should be conceptualized not simply as environmental influences but as markers of familial risk that may reflect inherited liability, environmental processes, and their interplay.

Maternal psychopathic traits represent a particularly relevant caregiver personality characteristic for understanding youth CU traits. Psychopathy is a multidimensional construct encompassing interpersonal manipulation, affective deficits, impulsive and irresponsible lifestyle features, and antisocial behavior (14, 15). Parental psychopathic traits have been associated with elevated CU traits in offspring (16, 17). However, it remains unclear whether specific psychopathy facets differentially relate to offspring CU traits. Notably, the affective facet of psychopathy, reflecting shallow affect and diminished empathy, is conceptually aligned with the socioemotional deficits central to CU traits and may therefore represent the psychopathy dimension most closely associated with offspring CU traits.

Most existing work on parental psychopathy and offspring CU traits has focused on childhood, with fewer studies examining adolescence and even fewer using designs that account for baseline CU traits when evaluating prospective associations. Further, many studies rely on global psychopathy scores, leaving unresolved whether specific psychopathy facets uniquely predict adolescent CU trajectories. Facet-level specificity is developmentally meaningful because interpersonal manipulation, antisocial behavior, and impulsive lifestyle features may index behavioral deviance, whereas affective deficits capture core socioemotional impairments that may be more directly implicated in CU development.

In the current study, we addressed these gaps by testing whether maternal psychopathic traits prospectively predicted adolescent CU traits over a 12-month period in a sample of youth diagnosed with CD, controlling for baseline CU traits and demographic covariates (i.e., youth age, sex, race, and maternal age). Using the four-facet model of psychopathy (18), we examined the unique contributions of maternal interpersonal, affective, lifestyle, and antisocial psychopathic traits to adolescent CU trajectories. We hypothesized that maternal affective psychopathic traits would show the strongest prospective association with adolescent CU traits. Because developmental research suggests that associations between caregiver characteristics and offspring socioemotional functioning may differ by youth sex (9, 19), we also examined whether youth sex moderated associations between maternal psychopathic traits and adolescent CU trajectories.

## Methods

### Participants

The analytic sample included 99 youth–caregiver dyads (*N* = 198 individuals). Youth were between 13 and 17 years of age (*M* = 15.52, *SD* = 1.31) and met diagnostic criteria for CD. Maternal caregivers ranged in age from 29 to 68 years (*M* = 44.53, *SD* = 8.15). The youth sample was predominantly male (68%) and racially identified as African American or Black (60%), Caucasian or White (36%), or American Indian or Alaska Native (4%). Participating mothers were predominantly biological mothers; thus, the racial composition of the youth sample approximated that of the participating mothers.

### Procedure

Participants were recruited through a large healthcare system in Virginia. Eligible youth had an existing diagnosis of CD established within the healthcare system by a licensed mental health professional before study enrollment. Prior to study procedures, youth and their caregivers were provided with a detailed study explanation and gave written assent and informed consent, respectively. Assent and consent were obtained separately in private settings to ensure confidentiality and minimize influence between participants. Data from the present analyses were drawn from a larger longitudinal project examining the role of fear reactivity in the development of CU traits (R01MH123535, PI: Thomson). Measures used in the present analyses were collected at baseline (Time 1) and at a follow-up assessment conducted 12 months later (Time 2). Participants were compensated USD 200 for their participation in the broader study. The participating caregivers were primarily mothers, with only three fathers completing the assessments. Because of the small number of fathers, these cases were excluded to maintain analytic consistency and avoid combining heterogeneous caregiver groups. Consequently, analyses focused on maternal psychopathic traits. All study procedures were approved by the [Virginia Commonwealth University] Review Board and were conducted in accordance with National Institutes of Health research guidelines.

### Measures

#### Youth callous–unemotional traits

CU traits were measured using the Inventory of Callous–Unemotional Traits (ICU) (20), a widely used self-report measure of affective and interpersonal features associated with CU traits in youth. The ICU includes 24 items rated on a 3-point Likert scale (1 = not at all true to 3 = definitely true). The measure includes both positively and negatively worded items; negatively worded items were reverse scored such that higher scores reflect greater levels of CU traits. In the present study, the ICU demonstrated good internal consistency (*α* = .80). The ICU was administered at baseline and at the 12-month follow-up.

#### Maternal psychopathic traits

Maternal psychopathic traits were assessed using the Self-Report Psychopathy Scale–Short Form (SRP-SF) (21), a 28-item self-report instrument designed to assess four facets of psychopathy: Interpersonal (7 items; e.g., “I have pretended to be someone else in order to get something”), Affective (7 items; e.g., “I do not feel bad about hurting people”), Lifestyle (7 items; e.g., “I have often done something dangerous just for the thrill of it”), and Antisocial (7 items; e.g., “I have tricked someone into giving me money”). Items are rated on a 5-point Likert scale ranging from 1 (disagree strongly) to 5 (agree strongly), with higher scores indicating greater levels of psychopathic traits. In the present sample, the total scale and facet scores demonstrated good internal consistency (total scale *α* = .93; Interpersonal *α* = .83; Affective *α* = .77; Lifestyle *α* = .82; Antisocial *α* = .78).

### Data analysis plan

Hierarchical linear regression analyses were conducted to examine whether maternal psychopathic traits were associated with higher youth CU traits at 12-month follow-up, controlling for baseline youth CU traits, youth age, sex, race, and maternal age. In Step 1, baseline youth CU traits, youth age, sex, race, and maternal age were entered simultaneously. In Step 2, maternal total psychopathy was added to evaluate whether overall maternal psychopathic traits were associated with higher youth CU traits at 12 months. In Step 3, maternal psychopathy facet scores (interpersonal, affective, lifestyle, and antisocial) were entered to examine the unique contributions of specific psychopathic trait dimensions.

To examine whether associations between maternal psychopathic traits and youth CU traits differed by sex, a separate moderation model was estimated. Maternal psychopathy facet scores and interaction terms between sex and each psychopathy facet were entered simultaneously. Significant interactions were probed using simple slopes analyses.

All analyses were conducted in R (22). Continuous variables were mean centered prior to analyses. Incremental model fit was evaluated using changes in variance (*ΔR*^2^) and nested model comparison. Regression tables report both unstandardized coefficients (*b*) with their standard errors (*SE*) and standardized regression coefficients (*β*). Multicollinearity was assessed using predictor-level variance inflation factors (VIFs).

## Results

### Descriptive statistics and correlations

Descriptive statistics and bivariate correlations are presented in Table 1. Baseline CU traits were positively correlated with CU traits at the 12-month follow-up (*r* = .39, *p* < .001). Baseline CU traits were positively associated with maternal total psychopathy (*r* = .23, *p* = .024), interpersonal psychopathic traits (*r* = .21, *p* = .039), and affective psychopathic traits (*r* = .22, *p* = .031). Follow-up CU traits at 12 months were positively correlated with maternal total psychopathy (*r* = .27, *p* = .007) and affective psychopathic traits (*r* = .35, *p* < .001). Consistent with expectations, maternal psychopathy facets were moderately to strongly intercorrelated (*r* = .61 to *r* = .69, *p*'s < .001).

**Table 1:** Correlations for main study variables.

	1	2	3	4	5	6	7	8	9
1. Youth CU traits (T1)	–								
2. Youth CU traits (T2)	.39***	–							
3. Maternal Psychopathy	.23*	.27**	–						
4. Maternal Interpersonal	.21*	.17	.88***	–					
5. Maternal Affective	.22*	.35***	.89***	.69***	–				
6. Maternal Lifestyle	.15	.21	.83***	.61***	.65***	–			
7. Maternal Antisocial	.19	.19	.83***	.69***	.65***	.63***	–		
8. Youth age	.03	.16	.00	.04	.04	−.05	−.05	–	
9. Maternal age	−.17	−.15	−.15	−.08	−.18	−.10	−.16	−.01	–
*M*	26.79	29.52	38.67	9.39	10.25	9.49	8.39	15.52	44.53
*SD*	9.32	9.06	12.74	3.76	4.16	3.73	2.71	1.31	8.15

CU, callous-unemotional; T1, baseline; T2, follow-up at 12 months.

*p < .05.

**p < .01.

***p < .001.

### Hierarchical regression analyses

Results from the hierarchical regression analyses are presented in Table 2.

**Table 2:** Main effects model.

Predictor	*B*	*SE*	β	*R* ^2^
Step 1				.21*
Youth Age	1.06	0.66	.15	
Youth Sex	3.14	1.89	.16	
Youth Race	0.06	1.84	.00	
CU traits (T1)	0.37	0.10	.35***	
Maternal age	−0.08	0.11	−.08	
Step 2				.26*
Youth Age	1.06	0.65	.15	
Youth Sex	3.91	1.85	.20*	
Youth Race	−0.78	1.76	−.04	
CU traits (T1)	0.32	0.11	.31**	
Maternal age	0.01	0.00	.11	
Maternal Psychopathy	0.16	0.07	.22*	
Step 3				.31*
Youth Age	1.01	0.64	.14	
Youth Sex	3.96	1.85	.20*	
Youth Race	−0.68	1.86	−.04	
CU traits (T1)	0.32	0.11	.29**	
Maternal age	−0.01	0.11	−.01	
Maternal Interpersonal	−0.41	0.35	−.16	
Maternal Affective	0.91	0.32	.41**	
Maternal Lifestyle	0.07	0.33	.03	
Maternal Antisocial	−0.06	0.47	−.02	

CU, callous-unemotional.

* *p* < .05.

** *p* < .01.

*** *p* < .001.

In Step 1, baseline CU traits, youth age, sex, race, and maternal age were entered. The overall model was significant, *F*(5, 93) = 4.71, *p* < .001, *R*^2^ = .21. Higher baseline CU traits, *b* = 0.37, *SE* = 0.10, *t* = 3.63, *p* < .001, and male sex, *b* = 3.91, *SE* = 1.85, *t* = 2.11, *p* = .037, were associated with higher CU traits at 12 months. Youth age, sex, race, and maternal age were not significant predictors, *p*'s ≥ .101.

In Step 2, maternal total psychopathy was added and significantly improved model fit, *ΔR*^2^ = .06, *F*(1,93) = 5.67, *p* = .019. The overall model was significant *F*(6, 93) = 5.54, *p* < .001, *R*^2^ = .26. Higher baseline CU traits, *b* = 0.32, *SE* = 0.11, *t* = 3.18, *p* = .002, male sex, *b* = 3.91, *SE* = 1.85, *t* = 2.11, *p* = .037, and higher maternal psychopathy, *b* = 0.16, *SE* = 0.07, *t* = 2.38, *p* = .019, were associated with higher youth CU traits at 12-month follow-up.

In Step 3, maternal psychopathy facet scores were entered simultaneously. The addition of the psychopathy facets significantly improved model fit, *ΔR*^2^ = .09, *F*(4,89) = 2.97, *p* = .024. The overall model remained significant, *F*(9, 89) = 4.16, *p* < .001, *R*^2^ = .31. Of the four psychopathy facets, only maternal affective psychopathic traits were significantly associated with youth CU traits at 12-month follow-up, *b* = 0.91, *SE* = 0.32, *t* = 2.85, *p* = .005. Maternal interpersonal, lifestyle, and antisocial psychopathic traits were not significant predictors (*p*'s ≥ .255).

### Moderation by youth sex

**Figure 1:**
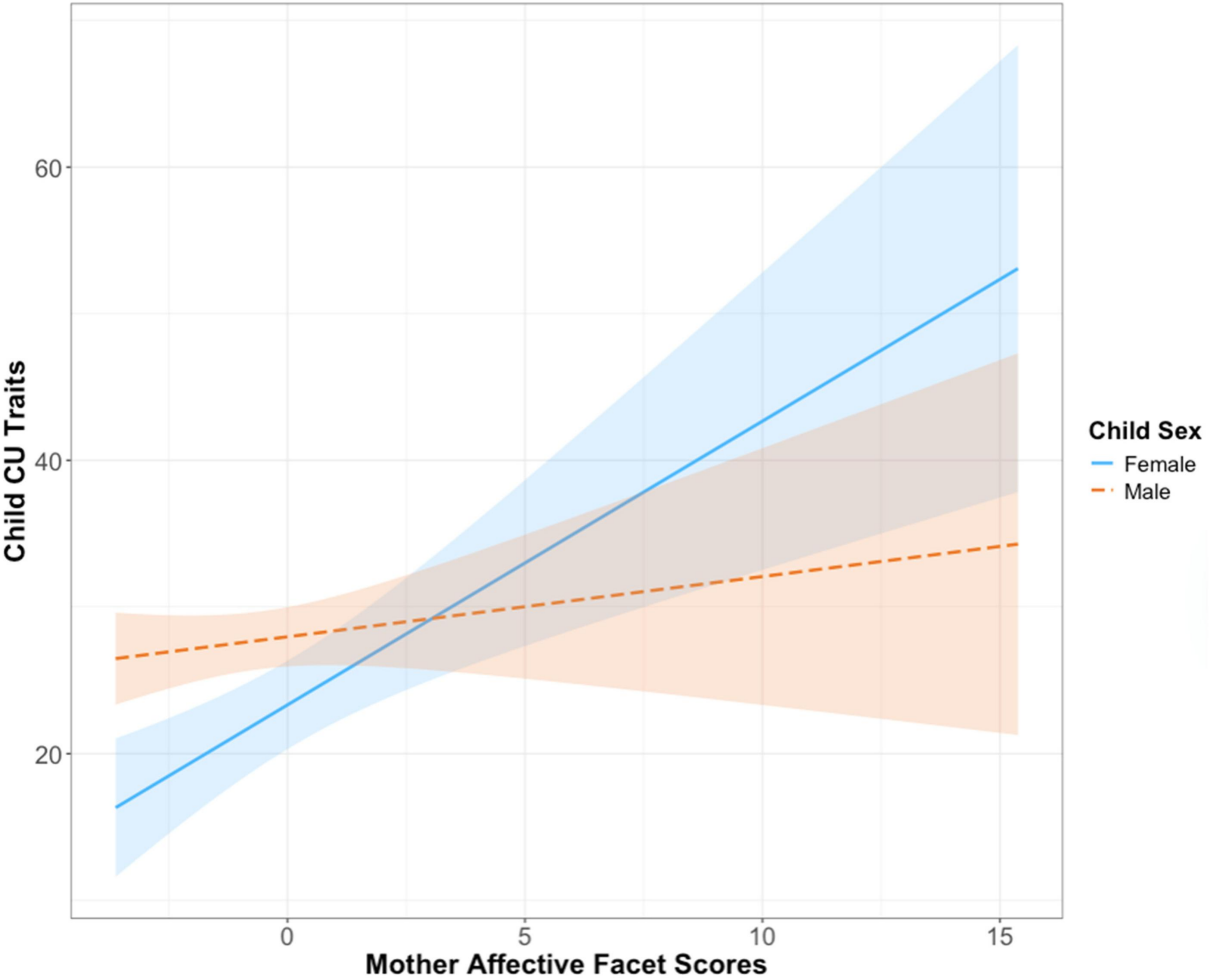
Maternal affective facet and youth CU traits by sex.

Results from the moderation analyses are presented in Table 3. Interaction terms between youth sex and each maternal psychopathy facet were entered simultaneously. The addition of the interaction terms did not significantly improve model fit, *ΔR*^2^ = .057, *F*(4,85) = 1.86, *p* = .124. The overall model was significant, *F*(13, 85) = 3.56, *p* < .001, *R*^2^ = .35. The interaction between youth sex and maternal affective psychopathic traits was significant, *b* = −1.47, *SE* = 0.63, *t* = −2.33, *p* = .022. Simple slopes analyses indicated that maternal affective psychopathic traits were associated with higher youth CU traits among girls, *b* = 1.81, *SE* = 0.48, *t* = 3.75, *p* < .001, but not among boys, *p* = .421 (Figure 1). No other interaction terms were significant (*p*'s ≥ .065).

**Table 3:** Moderation model.

Predictor	*B*	*SE*	β	*R^2^*
Moderation Model				.35*
Youth Age	0.83	0.64	.12	
Youth Sex	4.15	1.82	.21*	
Youth Race	−0.13	1.87	-.01	
CU traits (T1)	0.29	0.11	.27**	
Maternal age	−0.05	0.11	−.04	
Maternal Interpersonal	−0.48	0.58	−.19	
Maternal Affective	1.81	0.48	.81***	
Maternal Lifestyle	−0.91	0.64	−.36	
Maternal Antisocial	−0.32	0.92	−.09	
Sex × Interpersonal	0.19	0.73	.06	
Sex × Affective	−1.47	0.63	−.49*	
Sex × Lifestyle	1.35	0.72	.43	
Sex × Antisocial	0.53	1.07	.12	

CU, callous-unemotional.

* *p* < .05.

** *p* < .01.

*** *p* < .001.

## Discussion

The present study examined whether maternal psychopathic traits were prospectively associated with youth CU traits over 12 months in adolescents with CD. Consistent with prior longitudinal findings (16, 17), maternal total psychopathy was associated with higher youth CU traits after accounting for baseline CU traits, youth age, sex, race, and maternal age. Extending prior research, facet-level analyses demonstrated that this association was primarily attributable to the affective facet of psychopathy. Specifically, maternal affective psychopathic traits were uniquely associated with higher adolescent CU traits at follow-up, whereas interpersonal, lifestyle, and antisocial psychopathic traits were not independently associated with youth CU traits. These findings suggest that affective features of maternal personality, rather than broader interpersonal manipulation, impulsive lifestyle characteristics, and antisocial behavior, may be especially relevant for the persistence or escalation of CU traits during adolescence.

This facet-level specificity is theoretically meaningful. CU traits are characterized by core affective impairments, such as reduced empathy and limited emotional depth (2, 3). The affective facet of psychopathy similarly reflects impairments in empathy, guilt, and emotional responsiveness (15, 23). Although maternal total psychopathy was associated with higher youth CU traits at follow-up, the facet-level analyses indicated that this association was primarily driven by affective psychopathic traits. Prior research has demonstrated associations between parental psychopathic traits and offspring CU features (16, 17), but most studies have focused on total psychopathy scores. The present findings extend this literature by suggesting that maternal affective deficits may be particularly relevant for adolescent CU trajectories.

The present findings suggest that maternal affective psychopathic traits may represent an important caregiver personality characteristic associated with adolescent CU trajectories. This interpretation is consistent with developmental frameworks proposing that stable caregiver personality characteristics contribute to the broader family context in which children's socioemotional development unfolds (7, 8). Developmental theories emphasize that emotionally responsive caregiving is central to the development of conscience and empathy (19, 24), and previous research has consistently shown that caregiving characterized by low warmth, emotional disengagement, and harshness is associated with elevated CU traits across development (9, 25), highlighting the importance of the caregiving context for the emergence and persistence of CU traits. Although parenting processes were not directly assessed in the present study, our findings extend this literature by suggesting that caregiver personality characteristics may represent upstream familial risk markers that warrant consideration alongside parenting processes when examining adolescent CU trajectories. Individuals with elevated affective psychopathic traits characteristically exhibit reduced empathy, diminished perspective taking, and alexithymia (26), socioemotional characteristics that developmental theories suggest are important for scaffolding empathy development and moral internalization (19, 27).

Rather than implying a specific caregiving mechanism, the present findings should be interpreted within contemporary developmental models emphasizing gene-environment interplay. CU traits are moderately to highly heritable (10), and maternal psychopathic traits may therefore function as indicators of familial risk that reflect inherited liability, passive gene-environment correlation, and environmental influences operating concurrently. Importantly, genetically informed studies demonstrate that genetic influences and caregiving environments are reciprocally interdependent, with responsive, warm caregiving buffering the expression of genetic risk for CU traits and harsher caregiving amplifying the expression of genetic risk (9, 11, 12, 28). Consequently, maternal psychopathic traits should not be conceptualized solely as environmental risk factors or genetic proxies, but as caregiver characteristics that may index multiple interrelated developmental processes. Because parenting processes were not directly assessed, the present study cannot determine whether the observed associations reflect shared genetic liability, caregiving processes, passive gene-environment correlation, or interactions among these pathways. Future genetically informed longitudinal research incorporating direct measures of caregiver-child interactions will be important for disentangling these developmental processes and clarifying the pathways through which caregiver personality characteristics contribute to adolescent CU trajectories.

Although the overall interaction block did not significantly improve model fit, a significant interaction emerged between youth sex and maternal affective psychopathic traits such that the pattern of simple slopes was associated with higher CU traits among girls. This pattern suggests that associations between caregiver personality characteristics and adolescent CU traits may differ by youth sex. Developmental research suggests that girls' socioemotional functioning may be more closely influenced by relational and affective family processes than boys' externalizing pathways (9, 19). Although replication is needed, these findings raise the possibility that maternal affective dispositions may operate differently across sons and daughters during adolescence. Importantly, because the present study included only mother-child dyads, it is also possible that these findings reflect sex-specific dynamics within mother-daughter relationships rather than greater female sensitivity more broadly. Future studies incorporating both maternal and paternal caregivers will be important for determining whether these associations vary as a function of caregiver sex, youth sex, or their interaction.

An important contribution of the present study is demonstrating that caregiver personality characteristics remain relevant during adolescence. Much of the existing literature linking parental psychopathy with offspring CU traits has focused on early and middle childhood (7, 29–31). Adolescence is characterized by increasing autonomy and consolidation of personality traits (32, 33), yet it remains a period of continued relational sensitivity and socioemotional development (34, 35). The present findings suggest that maternal affective personality characteristics may continue to be associated with CU trajectories during this developmental window, even after accounting for substantial stability in CU traits over time. These findings therefore extend developmental models of CU traits beyond childhood by highlighting adolescence as a developmental period during which caregiver personality characteristics remain relevant to individual differences in CU trajectories.

### Clinical implications

CU traits are associated with reduced treatment responsiveness and elevated long-term risk (36). Meta-analytic evidence suggests that interventions incorporating caregiver components are generally more effective than youth-focused approaches alone for disruptive behavior disorders (37). However, many interventions emphasize behavior management while giving comparatively less attention to caregiver socioemotional characteristics (38). The present findings suggest that assessment of caregiver personality characteristics, particularly affective psychopathic traits, may provide clinically meaningful information when conceptualizing familial risk for adolescents with CU traits. Although the present study does not identify specific intervention targets or mechanisms, consideration of caregiver socioemotional characteristics may help inform individualized case formulation and guide future efforts to determine whether interventions that support caregivers' socioemotional functioning improve outcomes for youth with elevated CU traits.

### Strengths and limitations

Strengths of this study include a sample of adolescents diagnosed with CD, a prospective design controlling for baseline CU traits, and facet-level modeling of maternal psychopathy. Several limitations should also be considered. First, maternal psychopathic traits were assessed via self-report, and paternal caregivers were not included, limiting the generalizability of the findings. Second, the present study did not include direct measures of parenting behaviors or broader caregiving processes, precluding examination of the mechanisms through which caregiver personality may be associated with adolescent CU traits. Third, although we considered the findings within a gene-environment framework, the study design could not disentangle shared genetic liability, passive gene-environment correlation, and environmental influences. Future genetically informed, multi-method longitudinal studies incorporating observational and multi-informant assessments of caregiver-child interactions will be important for clarifying these developmental pathways. Finally, the present study did not examine youth exposure to childhood maltreatment, which may contribute to the development and maintenance of CU traits. Future longitudinal research should examine the extent to which trauma-related experiences mediate or moderate the associations between caregiver personality characteristics and youth outcomes.

## Conclusions

Maternal affective psychopathic traits were prospectively associated with higher levels of adolescent CU traits at 12-month follow-up in a sample of youth diagnosed with CD. These findings suggest that caregiver personality characteristics, particularly affective psychopathic traits, represent important familial risk markers associated with adolescent CU trajectories. By extending developmental models beyond parenting processes alone, the present findings highlight the importance of considering caregiver personality characteristics when examining adolescent CU traits. Future research integrating caregiver personality characteristics, direct assessments of caregiving, and genetically informed methodologies will be essential for refining developmental models of CU traits and clarifying the mechanisms through which caregiver characteristics and caregiving processes jointly contribute to developmental pathways associated with CU traits.

## Data Availability

Due to the sensitive nature of the clinical data and participant confidentiality, the dataset is not publicly available. De-identified data may be shared upon request and subject to institutional and IRB approval. All analysis code is available from the corresponding author upon request. Study materials and measures are available upon request, except where restricted by copyright or licensing. Requests to access the datasets should be directed to nicholas.thomson@vcuhealth.org.

## References

[1] American Psychiatric Association. Diagnostic and Statistical Manual of Mental Disorders: DSM-5-TR. American Psychiatric Associations (2022).

[2] Fairchild G Hawes DJ Frick PJ Copeland WE Odgers CL Franke B. Conduct disorder. Nat Rev Dis Primers. (2019) 5:1–25. 10.1038/s41572-019-0095-y 31249310

[3] Frick PJ Ray JV Thornton LC Kahn RE. Can callous-unemotional traits enhance the understanding, diagnosis, and treatment of serious conduct problems in children and adolescents? A comprehensive review. Psychol Bull. (2014) 140:1–57. 10.1037/a0033076 23796269

[4] World Health Organization. International Statistical Classification of Diseases and Related Health Problems (2019).

[5] Thomson ND Kjaervik SL Kaouar S Kimonis ER. Parenting under pressure: how child limited prosocial emotions shape the stress-warmth connection. Res Child Adolesc Psychopathol. (2025) 53:1397–407. 10.1007/s10802-025-01338-6 40569541 PMC12423222

[6] Frick PJ Ray JV Thornton LC Kahn RE. Annual research review: a developmental psychopathology approach to understanding callous-unemotional traits in children and adolescents with serious conduct problems. J Child Psychol Psychiatry. (2014) 55:532–48. 10.1111/jcpp.12152 24117854

[7] Belsky J. The determinants of parenting: a process model. Child Dev. (1984) 55:83–96. 10.2307/1129836 6705636

[8] Prinzie P Stams GJJM Deković M Reijntjes AHA Belsky J. The relations between Parents’ big five personality factors and parenting: a meta-analytic review. J Pers Soc Psychol. (2009) 97:351–62. 10.1037/a0015823 19634980

[9] Hyde LW Waller R Trentacosta CJ Shaw DS Neiderhiser JM Ganiban JM. Heritable and nonheritable pathways to early callous-unemotional behaviors. Am J Psychiatry. (2016) 173:903–10. 10.1176/appi.ajp.2016.15111381 27056607 PMC5008992

[10] Moore AA Blair RJ Hettema JM Roberson-Nay R. The genetic underpinnings of callous-unemotional traits: a systematic research review. Neurosci Biobehav Rev. (2019) 100:85–97. 10.1016/j.neubiorev.2019.02.018 30817934 PMC6756755

[11] Henry J Dionne G Viding E Vitaro F Brendgen M Tremblay RE. Early warm-rewarding parenting moderates the genetic contributions to callous–unemotional traits in childhood. J Child Psychol Psychiatry. (2018) 59:1282–8. 10.1111/jcpp.12918 29683187

[12] Tomlinson RC Hyde LW Dotterer HL Klump KL Burt SA. Parenting moderates the etiology of callous-unemotional traits in middle childhood. J Child Psychol Psychiatry. (2022) 63:912–20. 10.1111/jcpp.13542 34796486 PMC10049759

[13] Todorov JJ Devine RT De Brito SA. Association between childhood maltreatment and callous-unemotional traits in youth: a meta-analysis. Neurosci Biobehav Rev. (2023) 146:105049. 10.1016/j.neubiorev.2023.105049 36681371

[14] Hare RD Harpur TJ Hakstian AR Forth AE Hart SD Newman JP. The revised psychopathy checklist: reliability and factor structure. Psychol Assess. (1990) 2:338–41. 10.1037/1040-3590.2.3.338

[15] Neumann CS Hare RD Newman JP. The super-ordinate nature of the psychopathy checklist-revised. J Pers Disord. (2007) 21:102–17. 10.1521/pedi.2007.21.2.102 17492916 PMC3136810

[16] Dotterer HL Burt SA Klump KL Hyde LW. Associations between parental psychopathic traits, parenting, and adolescent callous-unemotional traits. Res Child Adolesc Psychopathol. (2021) 49:1431–45. 10.1007/s10802-021-00841-w 34152500 PMC8455443

[17] Ručević S Farrington DP Andershed H. The role of parental psychopathic traits: longitudinal relations with parenting, child’s psychopathy features and conduct problems. Curr Psychol. (2023) 42:23045–58. 10.1007/s12144-022-03452-w

[18] Hare RD Neumann CS. Psychopathy as a clinical and empirical construct. Annu Rev Clin Psychol. (2008) 4:217–46. 10.1146/annurev.clinpsy.3.022806.091452 18370617

[19] Kochanska G Aksan N Knaack A Rhines HM. Maternal parenting and children’s conscience: early security as moderator. Child Dev. (2004) 75:1229–42. 10.1111/j.1467-8624.2004.00735.x 15260874

[20] Kimonis ER Frick PJ Skeem JL Marsee MA Cruise K Munoz LC. Assessing callous–unemotional traits in adolescent offenders: validation of the inventory of callous–unemotional traits. Int J Law Psychiatry. (2008) 31:241–52. 10.1016/j.ijlp.2008.04.002 18514315

[21] Paulhus DL Neumann CS Hare RD. Manual for the Self-Report Psychopathy Scale. Ontario, CA: Multi-Health Systems (2016).

[22] R Core Team. R: A Language and Environment for Statistical Computing. Vienna, Austria: R Foundation for Statistical Computing (2025).

[23] Thomson ND. Understanding Psychopathy: The Biopsychosocial Perspective. New York: Routledge (2019).

[24] Craig SG Dawson A Chen S Moretti MM Pepler DJ. A systematic review of callous-unemotional traits and attachment in children and adolescents. Attach Hum Dev. (2024) 26:133–58. 10.1080/14616734.2024.2349569 38704613

[25] Waller R Trentacosta CJ Shaw DS Neiderhiser JM Ganiban JM Reiss D. Heritable temperament pathways to early callous–unemotional behaviour. Br J Psychiatry. (2016) 209:475–82. 10.1192/bjp.bp.116.181503 27765772 PMC5152869

[26] Burghart M Mier D. No feelings for me, no feelings for you: a meta-analysis on alexithymia and empathy in psychopathy. Pers Individ Dif. (2022) 194:111658. 10.1016/j.paid.2022.111658

[27] Cooke JE Stuart-Parrigon KL Movahed-Abtahi M Koehn AJ Kerns KA. Children’s emotion understanding and mother–child attachment: a meta-analysis. Emotion. (2016) 16:1102–6. 10.1037/emo0000221 27606827

[28] Waller R Hyde LW Klump KL Burt SA. Parenting is an environmental predictor of callous-unemotional traits and aggression: a monozygotic twin differences study. J Am Acad Child Adolesc Psychiatry. (2018) 57:955–63. 10.1016/j.jaac.2018.07.882 30522741 PMC6296820

[29] Loney BR Huntenburg A Counts-Allan C Schmeelk KM. A preliminary examination of the intergenerational continuity of maternal psychopathic features. Aggress Behav. (2007) 33:14–25. 10.1002/ab.20163 17441002

[30] Mendoza Diaz A Overgaauw S Hawes DJ Dadds MR. Intergenerational stability of callous–unemotional traits. Child Psychiatry Hum Dev. (2018) 49:480–91. 10.1007/s10578-017-0766-4 29119362

[31] Robinson BA Azores-Gococo N Brennan PA Lilienfeld SO. The roles of maternal psychopathic traits, maternal antisocial personality traits, and parenting in the development of child psychopathic traits. Parenting. (2016) 16:36–55. 10.1080/15295192.2016.1116894

[32] Goldstein BL Mackin DM Miao J Perlman G Watson D Ormel J. Is personality stable and symptoms fleeting? A longitudinal comparison in adolescence. J Res Pers. (2022) 97:104190. 10.1016/j.jrp.2022.104190 35241862 PMC8887882

[33] Soto CJ Tackett JL. Personality traits in childhood and adolescence: structure, development, and outcomes. Curr Dir Psychol Sci. (2015) 24:358–62. 10.1177/0963721415589345

[34] Allen JP Grande L Tan J Loeb E. Parent and peer predictors of change in attachment security from adolescence to adulthood. Child Dev. (2018) 89:1120–32. 10.1111/cdev.12840 28569384 PMC5711609

[35] Kilford EJ Garrett E Blakemore S-J. The development of social cognition in adolescence: an integrated perspective. Neurosci Biobehav Rev. (2016) 70:106–20. 10.1016/j.neubiorev.2016.08.016 27545755

[36] Thomson ND Perera RA Kevorkian S Hazlett L Vrana S. Impact VR: a socioemotional intervention for reducing CU traits, conduct problems, and aggression in youth with conduct disorder. Res Child Adolesc Psychopathol. (2025) 53:1865–78. 10.1007/s10802-025-01373-3 41055883 PMC12718285

[37] Perlstein S Fair M Hong E Waller R. Treatment of childhood disruptive behavior disorders and callous-unemotional traits: a systematic review and two multilevel meta-analyses. J Child Psychol Psychiatry. (2023) 64:1372–87. 10.1111/jcpp.13774 36859562 PMC10471785

[38] Thomson ND James JJ Blondell V Perera R Hazlett L Vrana S. Impact VR: building socioemotional resilience in youth with conduct disorder. Prev Sci. (2026) 27:250–65. 10.1007/s11121-025-01876-x 41535527 PMC12999750

